# Cell-Free Amniotic Fluid and Regenerative Medicine: Current Applications and Future Opportunities

**DOI:** 10.3390/biomedicines10112960

**Published:** 2022-11-17

**Authors:** Charles M. Bowen, Frederick S. Ditmars, Ashim Gupta, Jo-Anna Reems, William Samuel Fagg

**Affiliations:** 1 Department of Surgery, The University of Texas Medical Branch at Galveston, Galveston, TX 77555, USA; 2 John Sealy School of Medicine, The University of Texas Medical Branch at Galveston, Galveston, TX 77555, USA; 3 Future Biologics, Lawrenceville, GA 30043, USA; 4 BioIntegrate, Lawrenceville, GA 30043, USA; 5 South Texas Orthopaedic Research Institute (STORI Inc.), Laredo, TX 78045, USA; 6 Regenerative Orthopaedics, Noida 201301, UP, India; 7 Merakris Therapeutics, RTP Frontier 800 Park Offices Dr. Suite 3322, Research Triangle Park, NC 27709, USA; 8 Department of Engineering, University of Utah, Salt Lake City, UT 84112, USA; 9 Department of Biochemistry and Molecular Biology, The University of Texas Medical Branch at Galveston, Galveston, TX 77555, USA

**Keywords:** regenerative medicine, amniotic fluid, exosomes, tissue engineering, translational medicine

## Abstract

Amniotic fluid (AF) provides critical biological and physical support for the developing fetus. While AF is an excellent source of progenitor cells with regenerative properties, recent investigations indicate that cell-free AF (cfAF), which consists of its soluble components and extracellular vesicles, can also stimulate regenerative and reparative activities. This review summarizes published fundamental, translational, and clinical investigations into the biological activity and potential use of cfAF as a therapeutic agent. Recurring themes emerge from these studies, which indicate that cfAF can confer immunomodulatory, anti-inflammatory, and pro-growth characteristics to the target cells/tissue with which they come into contact. Another common observation is that cfAF seems to promote a return of cells/tissue to a homeostatic resting state when applied to a model of cell stress or disease. The precise mechanisms through which these effects are mediated have not been entirely defined, but it is clear that cfAF can safely and effectively treat cutaneous wounds and perhaps orthopedic degenerative conditions. Additional applications are currently being investigated, but require further study to dissect the fundamental mechanisms through which its regenerative effects are mediated. By doing so, rational design can be used to fully unlock its potential in the biotechnology lab and in the clinic.

## 1. Introduction

Some of the earliest documented accounts of using amniotic fluid (AF) as a therapeutic treatment date back to the dawn of the 20th century [1,2,3,4,5]. Among these are reports of using AF to minimize postoperative peritoneal adhesions and to treat orthopedic conditions [2,3,4]. These early studies paved the way for investigators to test the usefulness of AF for the treatment of other conditions, several of which yielded positive outcomes. Consequently, the diverse therapeutic potential of AF has led to an increase in fundamental, translational, and clinical research endeavors over the last decade [6,7]. These range from investigating its use in treating complicated wounds and ulcers, to treating complex pathologies such as osteoarthritis. The abundance, accessibility, safety profile, and regenerative potential of AF make it an attractive remedy for treating many human diseases/disorders. Accordingly, there is a high level of interest in determining the full spectrum of its uses in biotech and clinical applications.

AF is a complex biofluid whose composition changes throughout pregnancy. The production of AF occurs within two finite periods of embryogenesis: early and late gestation [8]. During early gestation, AF is primarily composed of water, electrolytes, and proteins that diffuse from maternal serum through aquaporin channels embedded along the amnion and chorion [9]. In the late gestational period, AF becomes more similar to fetal extracellular fluid primarily due to the production of fetal urine (600 to 1200 mL/day) and pulmonary secretions (60 to 100 mL/kg fetal body weight/day) [10,11]. In addition to its primary biological function, which is to support fetal growth and development, AF physically protects the fetus from external forces and trauma sustained by the mother, prevents umbilical cord compression between the fetus and uterine wall, and lubricates the fetal-maternal interface to prevent friction in utero [12].

**Figure 1:**
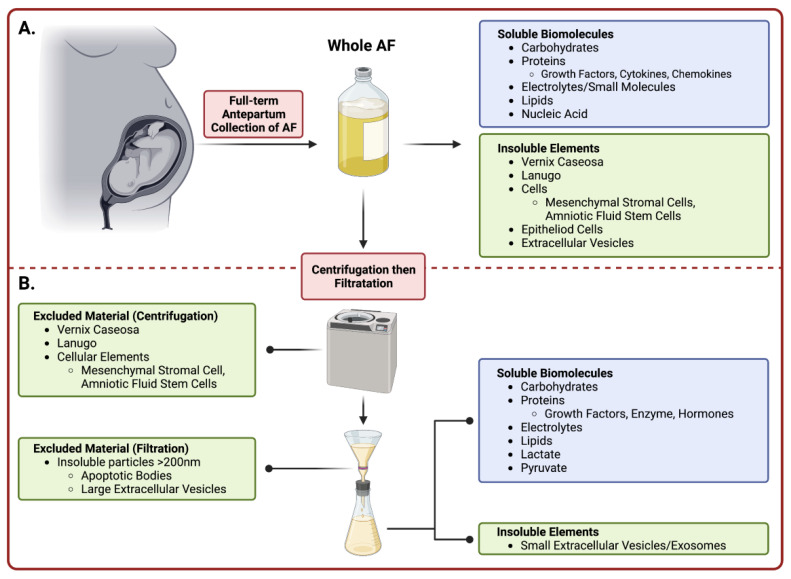
Summary of Amniotic Fluid Collection and Processing. Amniotic fluid is collected during an elective Cesarean delivery prior to removal of the fetus. (**A**) The top panel denotes the constituents in whole amniotic fluid prior to processing. (**B**) The bottom panel (below dashed line) describes the preparatory stages of purifying whole amniotic fluid into cell-free amniotic fluid. Upon manual processing including centrifugation and subsequent filtration, the collected eluent is considered cell-free and comprised of vital soluble biomolecules and small insoluble EVs. (Image created with Bio Render).

This review focuses on full-term AF, which can be harvested using aseptic techniques during Cesarean births, requires minimal processing, and has been safely used in various regenerative medicine settings. It is composed of two major fractions, a soluble one and an insoluble fraction (Figure 1). The insoluble fraction of AF contains lanugo hair, vernix caseosa, extracellular vesicles (EVs) including exosomes, and a heterogenous population of cells [13]. The soluble fraction consists of water, electrolytes/small molecules, proteins/peptides, carbohydrates, lipids, nucleic acids, hormones and other metabolites that can be found freely in suspension or contained within EVs [13,14]. Some of the notable components that are likely to contribute to AF-associated regenerative effects are soluble growth factors (some of which promote organ system development in utero [13]), anti-inflammatory cytokines, and immunomodulatory chemokines [15,16,17]. Furthermore, antimicrobial peptides and immunoglobulins are present that protect the fetus from pathogens [18].

Prior to 31 May 2021, the commercialization of AF was assumed to be regulated under 21 CFR 1271 and Section 361 of the Public Health Service (PHS) act for manufacturing human cells, tissues and cellular and tissue-based products (HCT/Ps). Presumably, this meant that AF could be marketed and used to treat ailments if done so under “homologous use”. A consequence of this vague language was that AF was marketed and used to treat patients suffering from a variety of conditions, sometimes without any evidence-based support. Anecdotal reports from treatments with AF for wounds and osteoarthritis showed positive outcomes with no reported adverse events. However, the FDA released a comprehensive policy for managing HCT/Ps and regenerative medicine products in November 2017 which conveyed their intent to regulate AF as a drug under Section 351 of the PHS act. Accordingly, the FDA required that AF injectables be withdrawn from the market as of 1 June 2021 unless the commercial entity held an approved Biologic License Application (BLA) [19]. Consequently, some groups have redirected efforts towards submitting investigational new drug (IND) applications in order to perform clinical trials using AF for specific indications. This changing regulatory landscape is currently a hot topic in the biotechnology space, and care providers are urged to stringently evaluate claims and interface with the FDA to ensure compliance.

Although various formulations of AF have been prepared and tested for their regenerative properties [6,20,21,22], the purpose of this review is to discuss the uses, open questions, and challenges surrounding the therapeutic potential of full-term cell-free AF (cfAF). However, we do make brief comments on studies using other formulations and have clearly noted this when doing so. To be clear, cfAF is prepared by centrifuging and then filtering freshly isolated AF through either a 0.45 µm or a 0.2 µm filter (Figure 1). AF processed in this fashion are devoid of insoluble components (i.e., lanugo, vernix caseosa, EVs, cellular elements, etc.) larger than 450 or 200 nm (respectively), but retains its soluble components (i.e., electrolytes, proteins, peptides, carbohydrates, lipids, nucleic acids, hormones, and other metabolites) [23,24,25]. Additionally, we will highlight cutting-edge advancements in fundamental and translational studies, as well as ongoing clinical research regarding cfAF. Together, advancements in these areas support the notion that cfAF is a useful therapeutic agent that can restore, repair, and regenerate damaged tissues across various human conditions. While cfAF is the topic of this review, we acknowledge that there are a number of birth tissue derivatives (i.e., whole amniotic fluid, amniocytes, cord blood, amniotic and chorionic membrane, Wharton’s jelly, cord, and placental tissue), including conditioned media from placental cells [26] that are becoming increasingly recognized for their beneficial effects in the treatment of a variety of diseases/disorders. However, a discussion of these other birth-tissue derivatives is beyond the scope of this review, and we refer readers to Flores and colleagues for an excellent recent review [27].

## 2. AF Components: Biomolecules, Cells, and Extracellular Vesicles

**Figure 2:**
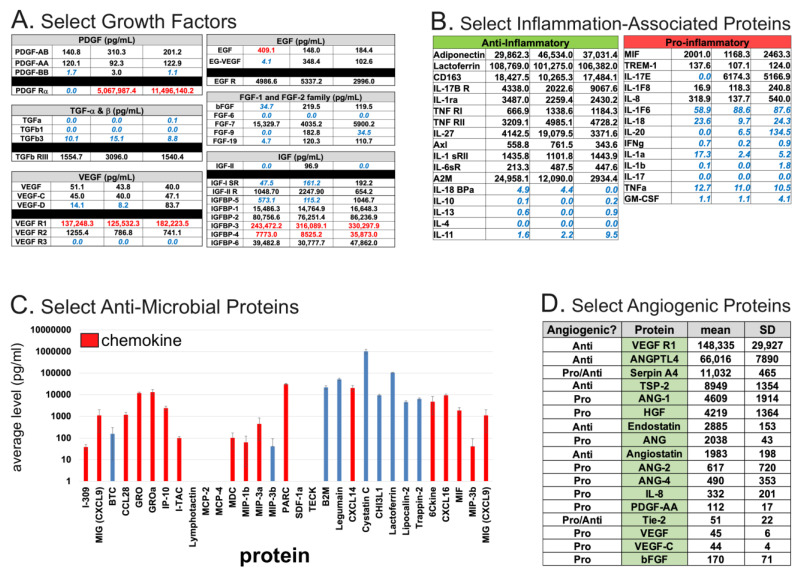
Summaries of select biomolecules of interest identified by multiplexed ELISA [15]. Each classification of the proteins shown was based on Gene Ontology annotations from the above-referenced study. (**A**). Select growth factors measured by ELISA from three independent donor-derived clinical grade cfAF samples. The growth factor is indicated by top header and left-most column, with the levels (pg/mL) measured indicated for each of three independent donors. Values in blue text indicated those below the level of detection for positive control, and those in red text indicate those above the level of detection for positive control. (**B**). Select inflammation-associated proteins measured as in part (**A**), and separated by anti-inflammatory (left) or pro-inflammatory (right) annotation. Values are shown in pg/mL and for each donor-specific batch of clinical grade cfAF as in (**A**). (**C**). Select mean values (pg/mL) of anti-microbial proteins shown by bar graph with error bars indicating standard deviation, as calculated in (**A**,**B**). Those shown in red are also annotated as chemokines. (**D**). Select angiogenic proteins shown, with “Angiogenic?” column header indicating whether the specific protein is annotated as pro- or anti-angiogenic, “Protein” column header indicating protein name, “mean” indicating mean value (pg/mL) from the three independent donor-derived lots of clinical grade cfAF as above, and “SD” indicating the standard deviation from the mean value.

The aforementioned biological functions of AF provide hints of its potential depth and breadth in the laboratory and clinical settings. Yet, due to the diversity and complexity of its components, comprehensive identification of its putative therapeutic factors (and their origins) has been challenging. However, recent advances in cell culture, cell sorting, and various -omics-based techniques have accelerated their discovery. Several studies have performed comprehensive metabolomic, proteomic, multiplex ELISA, or transcriptomic analyses that can identify specific components [15,17,28,29] that may promote regeneration. Given the different technical limitations of each of these approaches, combined with the immense complexity of the composition of cfAF, we urge caution before assigning any specific function(s) to a single component identified within it. For example, liquid chromatography with tandem mass spectrometry (LC-MS/MS) can readily identify the most abundant components in AF (such as Albumin), while those of lower abundance (but perhaps of greater importance to some readers) may be undetectable. In contrast, ELISA-based assays can be used to measure the levels of specific protein(s) of interest, as was done in clinical-grade cfAF derived from three independent donors [15]. We highlight the levels of select growth factors (Figure 2A), inflammatory-associated proteins (Figure 2B), anti-microbial proteins (some of which also have chemokine function; Figure 2C), and angiogenic proteins (Figure 2D) here, given their relevance in regenerative medicine. While one can conclude that clinical grade cfAF does contain groups of proteins associated with these various healing-related processes, we again urge tentative skepticism toward speculating about how a single component in AF might influence complex processes in cells and tissues, and identify this as a current gap in knowledge.

Various cell types and cellular elements have been identified in full-term AF. Most of these have an epithelial morphology and do not adhere to cell culture dishes or expand in vitro. Consequently, this has limited investigations into their secretory potential and contributions to cfAF components. In contrast, mesenchymal stromal cells (MSCs) are present in full-term AF, and hundreds of millions of amnion epithelial cells (AECs) line the amniotic cavity. Both of these cell types can be cultured in vitro and secrete abundant trophic factors with regenerative potential [30,31]. Amniotic fluid CD117/C-KIT positive stem cells (AFSCs) are another cell type present in AF (albeit at a lower abundance) that are more potent than MSCs [4], but do not form teratomas [32]. They also secrete trophic factors that likely contribute to the regenerative potential of AF [26]. Therefore, MSCs and AFSCs are the more well-characterized cells found in AF, and secrete trophic factors with regenerative potential.

EVs, which are found in the insoluble fraction of AF, are comprised of a lipid membrane housing various bioactive molecules that are secreted into the extracellular environment by all cell types [23,33]. These components facilitate intercellular communication via the exchange of nucleic acids and proteins [23,34], and play a role in both physiologic and pathologic contexts [23]. EVs are abundant in AF and are secreted from placental chorion and amnion, AF cells, and fetal cells of the pulmonary and gastrointestinal tracts [34]. Birth tissue EVs alone may have enormous regenerative potential, as a recent study indicated that EVs from neonatal umbilical cord (UC) confer regenerative effects to senescent bone marrow cells [34]. Many of the same factors from UC EVs are found in C-KIT+ AFSC-conditioned media, and also stimulate regenerative responses [26]. Taking these and other observations together, sourcing EVs from AF may be a promising alternative over cell-based biomanufacturing approaches. The latter also requires costly validation of donor cells’ stable genotypes and phenotypes, and optimization of biomanufacturing processes [35,36,37]. Despite the observations that AF EVs may have clear advantages compared to other sources of EVs, it is unclear how they can affect target cell/tissues, which warrants further investigation.

## 3. Fundamental and Translational Research Using cfAF

**Figure 3:**
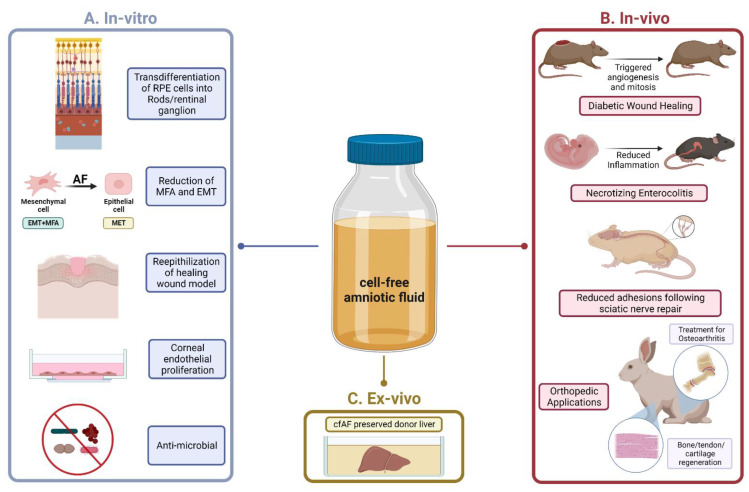
Summary of Translational Studies using Cell-Free Amniotic Fluid. (**A**). The in vitro studies (left panel) highlight fundamental studies that have added significant depth and understanding to the mechanistic underpinnings of how the therapeutic effects of cfAF are mediated in target cells/tissues. (**B**). The in vivo studies (right panel) highlight several animal models that have been used in cfAF research. Notable studies include rodent models of (from top to bottom): diabetic chronic wound healing, necrotizing enterocolitis, and nerve regeneration; rabbit models for various orthopedic applications including osteoarthritis and musculoskeletal regeneration are shown at the bottom. (**C**). The ex-vivo study (bottom, middle panel) references the application of cfAF in liver organ-tissue preservation prior to transplantation. (Image created with Bio Render).

Evidence that cfAF provides a therapeutic benefit to patients was first gleaned from fundamental research and pre-clinical studies using animal models (see Figure 3 for a summary). A notable early study found that cfAF significantly promoted the healing of diabetes-impaired wounds in rats, and the authors found that this was mediated through promoting mitosis and angiogenesis (see below; Figure 3) [38]. Cell-free AF also stimulates the re-epithelization of human skin in a wound healing model [39,40], promotes the in vitro trans-differentiation of retinal pigmented epithelial cells into rod photoreceptors and retinal ganglion cells [41], and stimulates the growth of human corneal endothelial cells (Figure 3) [42]. Moreover, cfAF contains sufficient components to protect the fetus via antimicrobial activity [43], and the soluble factors contained within cfAF act against a broad spectrum of wound-associated pathogens [44]. Similarly, another study used quantitative protein antibody arrays to examine full-term cfAF and found that the majority of the proteins detected have a role in host defense [15]. Together, these studies indicate that cfAF promotes wound healing, stem cell differentiation, cell growth, and has antimicrobial effects, each of which can contribute to its overall regenerative effects. While this begins to decipher some of the relevant cellular and molecular mechanisms involved in cfAF-based regenerative medicine, more fundamental studies are required to fully decipher how they are executed. These will include identifying and testing the role of specific biomolecules (or groups of them) within cfAF that are responsible for its regenerative properties.

Toward this goal, a recent report indicates that cfAF can effectively mitigate myofibroblast activation (MFA) and the epithelial-mesenchymal transition (EMT) in vitro, and begins to address the question about the biomolecules that elicit these functions (Figure 2) [17]. MFA is an underlying and causative factor in a plethora of disease states, particularly those associated with tissue fibrosis [45,46,47], so these findings have wide-ranging implications. This effect appears to be mediated, at least in part, by repressing TGFß signaling-based MFA and EMT. Intriguingly, EVs purified from cfAF were necessary and sufficient to activate MFA and EMT, and EV-depleted AF more potently repressed MFA and EMT [17]. Applying a similar approach to various models of degenerative conditions will illuminate the potential for using fractionated cfAF and could allow for precision medicine-based approaches to treating complex disease states. Below we summarize studies that have used cfAF as a treatment in various disease models.

### 3.1. Congenital Diseases

Necrotizing enterocolitis (NEC), a life-threatening inflammatory condition in premature infants, lacks durable interventions beyond complex surgical care, and bears a mortality rate of approximately 50 percent [24]. Moreover, surviving infants face developmental and gastrointestinal complications throughout their lifetime. Multiple sources of evidence suggest that AF can protect against the development of NEC, and one of which is that NEC is not observed in utero, when the fetus swallows significant amounts of AF [48]. Furthermore, data from rat NEC models indicate that AF-derived stem cell therapy improved survival through enhanced intestinal regeneration, which was driven by paracrine factors [49]. Further studies using EVs from AFSCs reported reduced intestinal injury and inflammation in rodent models of NEC [50,51]. These findings suggested that cfAF could also have protective effects in models of NEC. Indeed, a study conducted in fetal mice showed that microinjections of AF (n.b. it is unclear if the authors used cfAF or total AF) at gestational day 18.5 reduced LPS-mediated proinflammatory signaling in the gastrointestinal tract, which led to the decreased severity of NEC [52]. Also, in preterm pig models of NEC, treatment with enteral cfA, lowered the expression of inflammatory genes, decreased the abundance of bacterial microbes, and reduced NEC was observed [53,54]. These studies indicate that cfAF can effectively prevent and reduce the severity of NEC in various animal through its broad anti-inflammatory and anti-bacterial properties.

In addition to NEC, premature infants face a myriad of medical comorbidities and pre-term complications often arising from underdeveloped pulmonary function. Interventions such as surfactant therapy have greatly reduced mortality; however, the long term sequalae of chronic lung disease of prematurity affects one in every three extreme premature infants [55]. Bronchopulmonary dysplasia (BPD) is the most common pulmonary complication in premature infants, and severe cases may include pulmonary hypertension and right heart failure [56]. Many interventions have been applied to BPD, including stem cell and other cellular therapies; however, efficacy has been greatly limited by the common pitfalls of cell-based therapies such as poor cell viability, delivery, and scalability [25]. To circumvent some of these issues, investigators have successfully used AF MSC- or AFSC-conditioned media to reduce pulmonary fibrosis, abrogate alveolar damage, and promote regeneration in pulmonary hypoplasia [57,58,59,60]. These findings provided the rationale for a recent study that investigated the use of AF EVs to promote alveolarization and fibroblast maturation in BPD [25]. The authors discovered that AF EVs, which contain microRNAs correlated with anti-inflammatory and anti-oxidative stress activity, preserved lung alveolar development, decreased pulmonary hypertension, and reduced lung inflammation in rodent BPD models [25]. These findings suggest that the use of cfAF or its EVs could be effective reagents for treating premature infants that suffer from various conditions, which should be systematically tested in follow-up investigations.

### 3.2. Wound Healing

Early in fetal development, wounds and cutaneous lesions have the ability to repair without any trace of scar formation [61]. This concept has been well-documented and has led to focused studies exploring its molecular underpinnings and external driving factors. This is likely mediated in part by immersion of the fetus in amniotic fluid, which is rich in factors that promote repair and encourage regeneration [62]. Understanding how scarless fetal healing proceeds has been a major interest for burn/plastic surgeons and wound care specialists, thus investigating the use of cfAF in adult wound healing (and models thereof) may illuminate how scar reduction is mediated in these contexts.

AF has been used to treat complex wounds such as diabetic foot ulcers (DFU) and burn injuries. A critical aspect in their treatment is to restore lost or reduced blood supply by promoting angiogenesis [63,64]. The presence of pro-angiogenic proteins in clinical grade term cfAF [15] (Figure 2D), and its ability to promote angiogenesis in an in vitro assay [REF 15] suggests that this may be one of the mechanisms through which cfAF promotes wound healing. Burn and DFU wounds can also be prone to infection, which prevents healing, and may lead to limb amputation in patients with severe chronic wounds. A recent study discovered that cfAF retains its antimicrobial properties against a panel of broad-spectrum wound-associated pathogens (including *P. faecium*, *S. aureus*, *K. pneumonia*, *A. baumannii*, *P. aeruginosa*, and *E. aerogenes*), providing additional rationale for using cfAF in wound care [44]. However, the relevance and functional potency of this quality of cfAF remains to be directly tested in an animal model of wound. To test the efficacy of cfAF in models of wound healing, it was used to treat a rat model of chronic DFU, and it significantly accelerated the rate of wound closure. The authors showed that these effects were mediated, at least in part, by increased mitosis and angiogenesis in the rats treated with cfAF (Figure 3) [38], supporting the notion that cfAF promotes angiogenesis and may activate endogenous progenitor cell proliferation to stimulate healing. Furthermore, two different studies, one in a rabbit model of corneal wound healing and the other in fetal mice with cleft palate, independently observed accelerated reepithelization of the cornea or palatal edge, respectively, upon treatment with cfAF [65,66]. Finally, in a rat model of hernia repair, treatment with cfAF resulted in accelerated healing along with increased vascularity, collagen maturation, and re-epithelialization compared to controls [67]. Thus, cfAF can promote healing through stimulating angiogenesis and promoting re-epithelialization, and shows significant efficacy in treating wounds in animal models.

### 3.3. Nerve Repair

Microsurgical nerve repair faces many challenges such as epineural scar-forming adhesions and suboptimal nerve regeneration. A study in rats with surgically repaired sciatic nerves discovered that treatment with cfAF significantly reduced epineural scar tissue around the repair site, along with promoting improved functional outcomes and nerve fiber maturation (Figure 3) [68]. The data from this study indicate that cfAF provides positive neurotrophic and neurite-promoting factors that improve peripheral nerve regeneration following surgical anastomosis [68]. While additional pre-clinical studies may be required, these are promising results that suggest that AF may soon also be used in the clinic for particularly challenging cases of nerve repair or the prevention of nerve degeneration.

### 3.4. Musculoskeletal Applications

The use of AF in treating various animal models of musculoskeletal/orthopedic disorders has been extensively studied. Several studies have explored using cfAF to promote chondrogenesis from perichondral tissues to aid in the restoration of damaged tendons, ligaments, and joint spaces (Figure 2) [69,70]. Studies performed in both rats and rabbits used injections of cfAF [69,71] or amniotic suspension allograft (ASA, which consists of total AF and pulverized amnion) [72], to promote tissue and matrix grafting while limiting surgical adhesions following flexor tendon repair [69,71,72]. In a separate study, rabbits were treated with human cfAF following costal cartilage resection, and the authors found that those treated with cfAF showed improved chondrogenesis [73]. Together, these studies strongly suggest that cfAF can effectively stimulate the cellular events that contribute to joint regeneration.

Osteoarthritis (OA) is a degenerative disease that affects major joints such as the knee, and affects hundreds of millions of people worldwide [74]. Two recent studies indicated that treatment with ASA can effectively reduce OA symptoms in a rat model [74,75]. While this is not cfAF, a major component of ASA is liquid AF, thus cfAF may also reduce OA symptoms in this model.

cfAF has also been utilized in orthopedic spine research to determine if it can enhance vertebral spine fusions. A study performed in a rat model of vertebral fusions showed enhanced posterior spinal fusion in rats that received demineralized bone matrix (DBM) grafts enriched with cfAF compared to rats receiving DBM-only grafts [76]. Similar findings were also observed in a study using autografts enriched with cfAF in posterolateral spinal fusion: rats that received cfAF-enriched autografts had better outcomes compared to autograft-only controls [77]. These studies indicate that the addition of cfAF to graft components effectively promotes vertebral fusion.

Lastly, pre-clinical studies of fracture healing have demonstrated improved outcomes when using AF in the treatment regimen. In a rat tibial fracture model, those treated with cfAF showed improved healing as assessed via histological analysis of fracture-healing scores, suggesting that AF promotes bone remodeling and repair [77]. In a different study, cfAF was applied as a treatment following calvaria defects induced in rabbits, which showed increased bone ossification compared to saline controls [78]. These findings indicate that cfAF can also accelerate bone remodeling and repair following a fracture injury. The results of these studies together provide strong evidence that cfAF can reduce joint pain, promote overall joint health, and stimulate bone growth/healing, and therefore may be an effective treatment for various orthopedic conditions.

### 3.5. Other Translational Studies

Liver transplantation is replete with many challenges throughout the perioperative window, including difficulties with organ preservation. In fact, organ preservation upon resection and prior to transplantation to the recipient remains a major challenge for transplant surgeons. A recent study published an innovative method for static cold storage of the liver using cfAF (Figure 3) [79]. This comparative study observed that livers perfused with cfAF maintained organ viability to a level similar to the standard University of Wisconsin (UW) and Histidine-tryptophan-ketoglutarate (HTK) preservation solutions [79]. Although these limited analyses produced findings similar to standard of care methods, this is a rational approach that requires further investigation.

Ischemia reperfusion injury remains a major clinical concern in cardiology. Following myocardial infarct (MI), some cardiomyocytes fall into a reduced metabolic state [80]. After stenting the affected coronary vessels, these “hibernating” myocytes are flooded by oxygen rich blood, which generates cytotoxic free radicals and causes myocyte death [81]. In a study of rat myocardia, cfAF provided significant protection from reperfusion injury to “hibernating” myocardial cells [81]. While this also requires additional follow-up, it provides further evidence suporting the broad utility of cfAF in regenerative medicine.

## 4. Current Clinical Studies/Applications Using Cell-Free Amniotic Fluid or Derivatives

**Figure 4:**
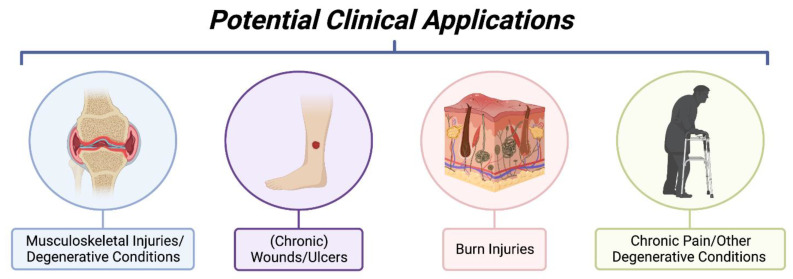
Summary of Potential Clinical Applications of Cell-Free Amniotic Fluid. Investigative clinical studies using cell-free amniotic fluid have been applied to musculoskeletal injuries such as those involving synovial joints, wound healing of chronic pressure ulcers, burn injuries to promote tissue regeneration, and in settings of chronic pain. The mentioned clinical applications are not exhaustive and only represent current applications under investigative study with published results. (Image created with Bio Render).

Given the evidence and studies described above, efforts were conducted to establish good-manufacturing-processes (GMP) to produce clinical-grade cfAF to treat patients. Prior to the FDA regulating AF as a drug, cfAF was therapeutically used to treat a variety of disorders in thousands of patients without any reports of adverse events. The following describes some of the published accounts of using cfAF in the clinic (summarized in Figure 4), while clinical studies or trials using cfAF or its derivatives are listed in Table 1 (completed studies/trials) or Table 2 (ongoing studies/trials).

### 4.1. Wound Healing

The use of amniotic fluid to encourage tissue repair and regeneration in challenging cutaneous wounds has recently yielded promising results in the clinic. Data from two case reports indicate that cfAF can effectively treat chronic wounds: (1) a persistent DFU was successfully treated by injection of cfAF [90]; and (2) two separate venous stasis ulcers affecting the same patient were successfully treated using a combination of cfAF injection around the wound bed with a topical treatment consisting of amniotic membrane. In the latter case, the wounds had persisted for two years despite correction of the underlying venous pathology and aggressive standard-of-care treatment [89]. The smaller of the two wounds closed after a single treatment, and the larger one did after two treatments [89]. This promising result is being followed-up with a phase 2 clinical trial; additional clinical trials using cfAF for wound treatment are also underway (see Table 2). Furthermore, a study consisting of 20 patients with chronic, treatment-resistant DFU assessed the use of ASA by injecting it into the wound bed, and then amniotic membrane was applied topically. Impressively, 90% of the patients experienced complete closure of their DFU within the first 12 weeks of treatment, which represented a significant improvement compared to traditional wound care methods. Moreover, all patients showed significant reductions in wound size, and no adverse events were reported [87].

Burn injuries can be a particularly difficult type of wound to treat, often requiring repeat treatments over several years to completely resolve. Even after successful treatment, burn wounds can leave extensive scarring that can impair quality of life and daily activities. Anecdotal reports suggest that AF and amniotic membrane are highly effective in treating burn wounds [91,92], but systematic clinical studies using standardized assays are needed to truly measure their efficacy. In pediatric patients, burn wounds can become chronic, and such cases are associated with high morbidity. In a 2021 retrospective study, four pediatric patients with chronic burn wounds treated with AF injections were identified and reviewed [93,94]. All of the patients’ treatment-resistant wounds closed following AF treatment, and once again, no adverse events were reported [94].

Finally, a pilot clinical study using autologous AF to aid in abdominal wound closure following cesarean delivery has been reported. This pilot study is ongoing and no data has been published yet, to the best of our knowledge (see Table 2).

### 4.2. Orthopedic Applications

Osteoarthritis affects over 30 million people in the United States, and therefore durable therapies remain a dire unmet clinical need. The mainstay therapy for refractory OA has been corticosteroid injections into the arthritic capsule or joint space. Such interventions provide reasonable relief of pain in the short-term, but these do not persist long-term. We note here that various different clinical studies using ASA report positive outcomes when treating OA patients, however we will not review these in depth since they do not test the use of AF/cfAF [86,95,96,97,98]. Given these encouraging results and those observed in animal models, however, cfAF may be an effective alternative capable of managing OA and other orthopedic conditions in patients.

A recent study reports the use of cfAF to manage chronic stenosing tenosynovitis (trigger finger), which was especially beneficial for patients with underlying diabetes, interestingly. In this study, 111 digits were injected with cfAF as a conservative intervention, and patients reported decreased pain, triggering, and improved disabilities of the arm, shoulder, and hand (DASH) scores compared to baseline measurements (Table 2) [85]. Additionally, several other ongoing studies using cfAF in an orthopedic setting exist (see Table 2), but have yet to publish any reports, to the best of our knowledge.

### 4.3. Ophthalmological Applications

Ocular injury is a stubborn and clinically complex problem due to the unique structure and immune privilege of the eye. Amniotic membranes (AM) have long been used to retain moisture and protect the eye against particulates that may interfere with the wound healing process [99]. Recently, this practice has expanded to include amniotic membrane extract and cfAF. Clinical reports show accelerated healing of the cornea after chemical burns [100], UVB radiation [101], stem cell damage [102] and other acute injuries [103] when treated with amniotic membrane extract. In a pilot study consisting of 22 patients with severe keratoconjunctivitis sicca (dry eye), cfAF drops were safe and more effective than traditional eye drops in reducing short term symptoms [104].

### 4.4. COVID-19

Given the evidence supporting the notion that cfAF is a safe anti-inflammatory biologic, several groups hypothesized that it might effectively protect against severe acute respiratory disease during SARS-CoV2 infection. Moreover, the total lack of any observed maternal to fetal transmission of COVID-19 suggested that placental tissues, including AF, may have a protective effect. One report describes a 10 patient study consisting of patients with hospital-diagnosed COVID-19: six patients received intravenous delivery of cfAF, which resulted in significantly improved morbidity and mortality, and no adverse effects [82]. Another group performed similar studies in COVID-19 patients with mild-moderate symptoms, also by injecting cfAF intravenously. The authors reported a significant reduction in inflammatory biomarker levels and other secondary markers of COVID-19 severity [84]. In another case study, three severely ill COVID-19 patients were treated with cAF and showed rapid and significant improvements in sequential organ failure assessment (SOFA) scores, ICU status, and respiratory function following treatment [88]. While these studies did demonstrate that cfAF treatments delivered intravenously for COVID-19 are feasible, safe, and potentially effective; higher-powered studies are needed to fully substantiate the efficacy of cfAF in the treatment of COVID-19.

## 5. Conclusions, Limitations, and Future Outlook

The studies reviewed here range from fundamental and translational investigations to clinical trials. Evidence from these studies support the notion that cfAF generally functions by reducing inflammation, modulating the immune response, promoting regeneration via new cell growth in situ, and returning tissue to its previous homeostatic resting state. In the laboratory and clinic, cfAF has been proven to be safe and shows efficacy in accelerating wound healing, treating musculoskeletal defects, treating nerve defects or post-operative nerve damage, as well as treating certain congenital defects. In the specific context of wound healing, the disparate studies discussed above converge on common themes: cfAF promotes angiogenesis, reduces pro-inflammatory gene expression patterns and signaling that are consistent with reducing MFA, and reduces EMT in favor of promoting re-epithelialization of cells/tissues. These effects are likely due to delivery of the various protein, lipid, and RNA components identified in cfAF (whether freely soluble or encapsulated in EVs) to the target cells/tissues; although further systematic studies into the precise and global effects on gene expression and cell signaling elicited by cfAF will be required to fully determine the extent to which this is occurs. These efforts will also have the potential to usher in a new era of precision medicine in various disease contexts. Despite these promising early results, cfAF is still relatively under-researched compared to cell- and stem cell-based regenerative approaches.

Several obstacles remain that limit cfAF’s role as a widely adopted therapeutic. At present, collecting AF during an elective Cesarean birth inherently limits supply. If it gains mainstream popularity this could hinder availability, but may also encourage more widespread collection and processing efforts. Some physicians are hesitant to use cfAF due to fear of causing AF embolism, despite several reports described here and elsewhere of its relatively safe intravenous use. In fact, recent evidence strongly suggests that AF embolism is likely caused by fetal antigens and not the thrombin present in AF [22]. Finally, natural variation in the makeup of AF between donors may have as yet uncharacterized effects on the therapeutic potential of cfAF [105,106,107]. Additional research is needed to evaluate these concerns and limitations.

Open questions remain about the components of cfAF and its uses, particularly around the basic mechanisms through which cfAF’s therapeutic effects are executed in target cells/tissues. Further fundamental studies are required to address these questions. Moreover, there are several applied studies in animal models that have yet to be tested in humans, including those treating reperfusion injury, fractures, congenital diseases, and scar reduction. By doing so, the field will move closer to fully evaluating cfAF and unlocking its full scope and potential in regenerative medicine.

## Figures and Tables

**Table 1:** Summary of Completed Clinical Trials using AF or AF-derived interventions.

Study	Condition	Reference/Group	Efficacy	Other Comments
“A pilot trial of human amniotic fluid for the treatment of COVID-19”	COVID-19	Selzman et al. 2021 [82,83] NCT04319731 Univ. of Utah	Higher dose patients had reduced C-reactive protein and improved clinical outcomes	Pilot study of 10 patients; 1 succumbed to COVID; last 6 patients received higher dose of AF; no AF-related AEs reported
“Proof-of-concept trial of an amniotic fluid-derived extracellular vesicle biologic for treating high risk patients with mild-to-moderate acute COVID-19 infection”	COVID-19	Bellio et al. 2022 [84] NCT04657406	Potentially effective use of cfAF to prevent severe disease progression in at-risk patients.	Pilot study of 8 patients; no serious AEs reported.
“Effectiveness of Amniotic Fluid Injection in the Treatment of Trigger Finger: A Pilot Study”	Stenosing tenosynovitis	Quinet et al. 2020 [85] NCT03583151 Athens Orthopedic Group	Half of patients noted improvements and did not receive alternative treatment.	Study included 111 digits from 96 patients with a significant reduction in pain, triggering/day, and DASH score. No AEs or complications discovered based on injection of AF. Study found AF helpful for patients with diabetes, a vulnerable population to tenosynovitis.
“A Randomized Controlled Single-Blind Study Demonstrating Superiority of Amniotic Suspension Allograft Injection Over Hyaluronic Acid and Saline Control for Modification of Knee Osteoarthritis Symptoms”	Osteoarthritis (OA) of the knee	Farr et al. 2019 [86] Knee Preservation and Cartilage Restoration Center; Hospital of Special Surgery; Rush Univ., NYU Lagnone Med., Organogenesis, Inc.	Demonstrated safety and trends towards improved pain and function.	Included 200 patients randomized 1:1:1 (Amniotic suspension:Hyaluronic Acid:saline)
“A prospective study of 20 foot and ankle wounds treated with cryopreserved amniotic membrane and fluid allograft”	Wound healing	Werber et al. 2013 [87]	May represent useful option to treat chronic diabetic foot wounds.	Clinical study using granulized amniotic membrane and fluid to treat chronic diabetic foot wounds in 20 patients. Patients were followed for 12-weeks with 90% (18/20 subjects) of wounds healed. None of the wounds (0/20) progressed to amputation.
“Case Report: Administration of Amniotic Fluid-Derived Nanoparticles in Three Severely Ill COVID-19 Patients”	COVID-19	Mitrani et al. 2021 [88]	Treatment using cfAF appeared to be safe in *n* = 3 patients.	No adverse events associated with therapy. All three patients developed respiratory failure with hospitalization greater than 40 days and showed improved clinical status while in the ICU via resolution of acute delirium and reduction of inflammatory biomarkers.

**Table 2:** Summary of Recruiting/On-going Clinical Trials using AF-derived interventions.

Study	Condition	Reference/Group	Efficacy	Other Comments
“Processed Amniotic Fluid (pAF) for the Treatment of Chronic Wounds”	Chronic refractory wounds	ClinicalTrials.gov (accessed on 17 October 2022) NCT04438174 Univ. of Utah	No results posted	Primary objective aims to determine safety and efficacy of using pAF to treat chronic wounds; 1 mL/5 cm^2^ direct wound injection; limited to two injections
“pAF for the Treatment of Osteoarthritis”	Osteoarthritis (OA) of the knee	ClinicalTrials.gov (accessed on 17 October 2022) NCT04886960 Univ. of Utah	No results posted	Randomized double-blinded standard of care (steroid) vs. sterile AF for OA; 3 mL, one time injection
“Sterile Amniotic Fluid Filtrate Epidural Injection”	Spinal Stenosis	ClinicalTrial.gov (accessed on 17 October 2022); NCT04537026 Univ. of Utah	No results posted	Double-blinded randomized prospective study of sterile AF filtrate epidural injection for treatment of lumbosacral radicular pain due to spinal stenosis
“The Use of Autologous Amniotic Fluid at Cesarean Wound Closure”	Wound healing	ClinicalTrials.gov (accessed on 17 October 2022) NCT04359472 Recibio, Inc. and Duke Univ.	Not reported	Collection and reapplication of AF to cesarean wound upon skin closure
“Processed Amniotic Fluid (PAF) Drops After Photorefractive Keratectomy (PRK)”	Photorefractive Keratectomy	ClinicalTrials.gov (accessed on 17 October 2022) NCT04281004 Univ. of Utah	Not Reported	Randomized, double-masked, placebo-controlled study to determine: safety of AF, rate of re-epithelialization, reduction in pain, vision improvements, and effects on ocular surface staining and corneal regularity.
“Study for the Treatment of Ocular Chronic Graft-Versus-Host Disease (GVHD) with Amniotic Fluid Eye Drops (AFED)”	Ocular Chronic Graft-Versus-Host Disease	ClinicalTrials.gov (accessed on 17 October 2022) NCT03298815 Univ. of Utah	Not reported	Randomized, double-blinded, placebo-controlled study assessing efficacy of processed AF for patients with hematologic malignancy who have received allogenic stem cell transplantation that develop chronic GVHD of the eye.
“Dermacyte Amniotic Wound Care Liquid for the Treatment of Non-healing Venous Stasis Ulcers”	Venous stasis ulcer	ClinicalTrials.gov (accessed on 17 October 2022) NCT04647240 Merakris Therapeutics	Case study demonstrated safety and efficacy in the treatment of chronic venous stasis ulcers with Dermacyte liquid (cfAF) and membrane in a 65-year old patient [89].	Randomized, double-blind, placebo controlled, two-part study. Part 1: 10 patients randomized 1:1 with Dermacyte Liquid (DL) once weekly or twice weekly to determine administration frequency for part 2. Part 2: 30 patients randomized 1:1 to receive DL or placebo (0.9% saline). Obtained FDA-approved IND.
“Efficacy of Amniotic Suspension Allograft in Patients with Osteoarthritis of the Knee”	Osteoarthritis (OA) of the knee	ClinicalTrials.gov (accessed on 17 October 2022) NCT04636229 Organogenesis	No results posted	Prospective, multicenter, randomized, double-blind, placebo-controlled Phase 3 study of ASA in patients with OA of the knee. Radom assignment (1:1) to receive either single intra-articular injection of 2 mL ASA (plus 2 mL saline) OR 4 mL normal saline. Estimated trial size of *n* = 474 subjects.

## Data Availability

This review article did not generate or report any new data.
